# Twisting Paths: The Paradox of Fiber Branching in Muscle Regeneration

**DOI:** 10.3390/ijms27020684

**Published:** 2026-01-09

**Authors:** Leonit Kiriaev, Kathryn N. North, Stewart I. Head, Peter J. Houweling

**Affiliations:** 1 Muscle Research Group, Murdoch Children’s Research Institute, Parkville, VIC 3052, Australia; 2 Department of Paediatrics, The University of Melbourne, Parkville, VIC 3010, Australia; 3 School of Medicine, Western Sydney University, Campbeltown, NSW 2560, Australia

**Keywords:** fiber branching, muscle regeneration, Duchenne muscular dystrophy, *mdx* mouse, eccentric contractions

## Abstract

Muscle regeneration following injury reveals a striking paradox: the same phenomenon, fiber branching, can serve as both a beneficial adaptation in healthy muscle and a pathological hallmark in disease. In healthy muscle, branched fibers emerge as an adaptive response to extreme mechanical loading, redistributing stress, enhancing hypertrophy, and protecting against injury. Conversely, in conditions such as Duchenne Muscular Dystrophy, excessive and complex branching contributes to mechanical weakness, increased susceptibility to damage, and progressive functional decline. This review explores the dichotomy of fiber branching in muscle physiology, synthesizing current research on its molecular and cellular mechanisms. By understanding the paradoxical nature of fiber branching, we aim to uncover new perspectives for therapeutic strategies that balance its adaptive and pathological roles to improve outcomes for muscle diseases.

## 1. Introduction

Skeletal muscle’s unique ability to regenerate after injury is a vital process that maintains muscle function and integrity following damage and relies on the coordinated activation of various cellular mechanisms including the breakdown of injured muscle fibers (necrosis), activation of muscle satellite/stem cells, and their proliferation and differentiation, followed by maturation of newly formed muscle fibers and remodeling of injured/damaged muscles back to a healthy state [1,2]. However, during this complex regenerative process, some fibers exhibit atypical remodeling in the form of branched fibers, which were initially thought to be due to incomplete myotube fusion. Importantly, it has been observed that in repetitive injuries, the degree of fiber branching increases with each round of injury, suggesting that skeletal muscle retains a “memory” of the number of past injuries [3,4]. This phenomenon, while sometimes adaptive, can also signal remodeling in response to pathological damage, as observed in progressive inherited muscle diseases such as Duchenne muscular dystrophy (DMD) [5,6]. Understanding the mechanisms that drive this imperfect fusion and the resulting fiber branching will be crucial in understanding disease pathobiology and uncovering new therapeutic strategies for the treatment of muscle diseases.

### Fiber Regeneration in Response to Injury and the Imperfect Fusion of Myogenic Cells

**Figure 1:**
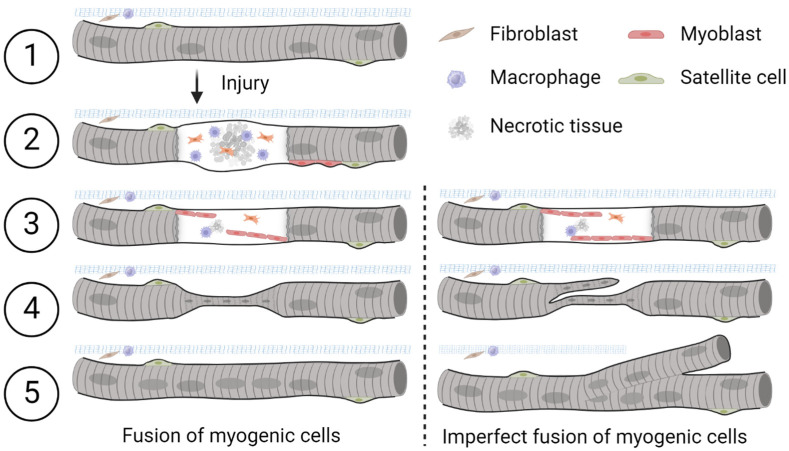
Schematic diagram of the damage repair process in a skeletal muscle fiber after injury. Following myofiber injury and necrosis, satellite cells are activated, proliferate, and fuse to form new or repair existing muscle fibers (**left**). Adapted from Dueweke et al. [7]. Imperfect lateral or longitudinal fusion of myogenic cells during regeneration may lead to the formation of branched muscle fibers (**right**). Satellite cells are muscle stem cells that, upon activation, can generate myoblasts for repair while also self-renewing to maintain the stem cell pool. Fibroblasts are cells responsible for laying down the extracellular matrix of connective tissues. Macrophages are immune cells that engulf damaged tissue and debris. (1) Healthy skeletal muscle. (2) Upon injury, satellite cells proliferate and differentiate into myoblasts along the periphery of the fiber. (3) Myoblasts align and stop proliferating. (4) Myoblasts fuse to form myotubes which connect ruptured ends of the fiber. Centralized nuclei appear during damage repair after myoblast fusion. (5) Repair is complete. This concept is illustrated here based on interpretations from multiple studies; schematic created by the authors.

Muscle has an innate ability to adapt to both mechanical strain and injury. Following damage, necrosis of individual muscle fibers may occur, which in turn triggers the activation of satellite cells to initiate regeneration. During the main stages of myogenesis and skeletal muscle repair, primitive satellite cells differentiate into muscle progenitor cells (myoblasts) that appear at the periphery of muscle fibers (Figure 1, left modified from Dueweke et al. [7]. Myoblasts fuse together to form primary myotubes containing centralized nuclei (an indicator of muscle regeneration). Later, secondary myotubes arise beneath the basal lamina with fusion of myoblasts radially and longitudinally (not seen in figure). Prior studies have highlighted and illustrated that, in transverse sections, primary and secondary myotubes can be visualized as a single cell, a cell in the process of longitudinal ‘division’ [8], or as two distinct cells during myogenesis [9]. In instances where they appear as distinct cells, secondary myotubes are differentiated from primary myotubes or myofibers by their smaller radial size. Over time, these secondary myotubes enlarge and mature to restore injured muscle to its pre-injury state [1,2].

A well-recognized phenomenon during muscle regeneration is that some myofibers appear to undergo longitudinal ‘division’ or ‘fusion’, leading to the formation of mid-section ‘fissures’ and ‘splits’ towards the ends of the fibers [10,11]. Over time, various terms have been used throughout the literature to describe these regenerated fibers, which exhibit atypical morphology. In this review, we adopt the term ‘branched’ to describe these complex and variably structured fibers, as originally suggested by Ontell and Feng [11]. However, to ensure clarity and consistency when referencing other studies, we will retain the specific terminology used by the original authors when citing their work.

A branched myofiber is defined as an isolated muscle fiber containing one or more offshoots or bifurcations along its length, all enclosed within a continuous sarcolemma. This encompasses fibers arising from imperfect fusion during regeneration, splitting-like formations, and other malformed fiber architectures observed across various conditions. While prior literature has used terms such as “split fibers” or “malformed fibers,” we follow the convention of Ontell and Feng [11] and Head [12] in using “branching” to describe the morphological outcome without implying a specific mechanism.

**Figure 2:**
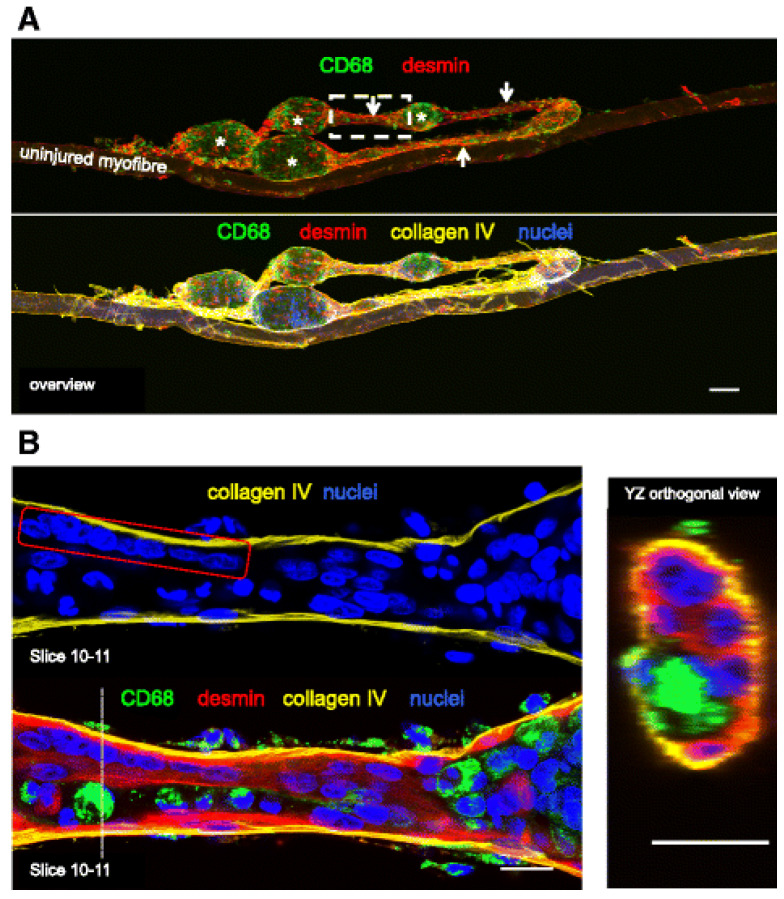
Myofilament formation at the basement membrane, Mackey and Kjaer [14]. (**A**) Confocal microscope image of a regenerating human muscle fiber (attached to an uninjured fiber), stained for desmin (red), macrophages (CD68, green), basement membrane (collagen IV, yellow) and nuclei (blue), 7 days post injury. The images are presented with all 4 colors merged or split into fewer channels for clarity. Note the distinct necrotic (asterisk) and regenerating (arrows) zones. Part of the fiber has become detached from the uninjured fiber and folded back on itself during the staining procedure. Note the capillaries stained with collagen IV on the surface of the fibers. Scale bar, 100 μm. The area indicated by the dashed line box was imaged at higher magnification, shown in (**B**). (**B**) Eight to nine adjoining nuclei can be seen (red box) in a desmin dense area of this regenerating zone, indicating they are potentially newly fused myogenic cells. Macrophages were also observed within the myofiber basement membrane in this narrow zone. The dashed line indicates the location of the YZ orthogonal view. Scale bars, 20 μm.

Branching is most likely a result from the incomplete fusion of myogenic cells during fiber segment regeneration after tissue necrosis [3,13]. A study performed by Mackey and Kjaer [14] induced myofiber injury on human vastus lateralis muscles through eccentric (lengthening) contractions, commonly employed in muscle physiology to induce damage [15]. The study found that, unlike undamaged muscles which typically contain a single myotube within each basement membrane (Figure 2), the repaired muscle fibers after regeneration contained multiple myotubes within the same basement membrane scaffold.

Incomplete fusion of myotubes during muscle regeneration can result in the formation of branched fibers [4]. This phenomenon, often arising from partial lateral fusion of regenerating fibers, has been observed not only in pathological conditions but also in non-diseased muscles undergoing regeneration [2,4]. These occurrences provide valuable insights into the dynamics of muscle fiber formation in adults and underscore the significance of fiber branching in the overall regenerative process. Additionally, further investigations by Mackey and Kjaer [14] show that muscle fibers with fissures frequently exhibit internal myonuclei located centrally within the fissure, indicative of ongoing cycles of degeneration and regeneration. These observations highlight the complexity of muscle repair mechanisms and suggest that continuous mechanical stress could lead to impaired regeneration, even in otherwise healthy muscle tissue.

## 2. Muscle Fiber Branching in Healthy Muscle

In non-dystrophic muscle, branched fibers are most commonly reported following regeneration induced by experimental injury [16,17] or extreme loading paradigms [18,19]. Branched fibers have also been observed in pathological contexts, including muscular dystrophy [11,20,21] and whole muscle transplants [22,23]. More recent studies have further explored the occurrence of fiber branching in non-dystrophic muscles under various stimuli, including extreme loading, ablation, electrical stimulation-induced injury and cardiotoxin/myotoxin-induced injury.

Together, these studies indicate that branched fibers can arise in non-dystrophic muscle following remodeling driven by injury and/or extreme loading. In many contexts, branching appears to reflect imperfect repair rather than a primary pathology, although its functional consequences likely depend on branch complexity and the mechanical demands placed on the muscle. This provides important context for later sections where branching becomes more frequent and maladaptive in dystrophic muscle.

While this section focuses on structural adaptations resulting from injury and overload paradigms, it is also important to acknowledge that fiber type may influence the propensity for branching. Fast- and slow-twitch fibers differ in their metabolic and mechanical properties, which may shape their responses to injury and subsequent regeneration. These distinctions are particularly relevant when considering the differing severity and frequency of branching observed in various muscle types [24,25]. Although this relationship is explored in more depth later in the context of dystrophic muscle, it remains incompletely defined in healthy muscle and warrants further investigation.

### 2.1. Response to Extreme Mechanical Loading

Recent studies suggest that branched fibers can form in response to extreme loading and hypertrophy in non- pathological (i.e., healthy) muscle tissue. The phenomenon has been observed across various animal models as described by Murach et al. [26] and, to a lesser extent, in humans [18,19]. In this section, we will review the various conditions under which branched muscle fibers form in response to extreme loading, drawing on evidence from various models including birds, rodents, cats and humans.

One of the most dramatic examples of mechanical loading leading to muscle hypertrophy and fiber branching is the wing-weighting model in birds. In this model, the accessory muscles of 26 week adult quails are subjected to stretch overload, leading to significant increases in muscle mass and fiber area. Antonio and Gonyea [27] reported a 111% increase in latissimus dorsi muscle fiber area and a 318% increase in muscle mass within 28 days, with muscle fiber number increasing by 82% on histological cross-sections. This increase was attributed to the longitudinal branching, fragmentation, or splitting of muscle fibers, occurring at the myofibril level, suggesting that extreme mechanical loading can induce muscle fiber branching without the presence of a marked degeneration-regeneration response [28].

The appearance of muscle fiber branching is a prominent feature observed in various resistance training paradigms across different animal models. In rats, weight-lifting exercises have shown significant muscle adaptations. Ho et al. [29] conducted a study where adult male albino rats were subjected to a progressive weight-lifting program, lifting loads equal to 130% of their body weight. This regimen resulted in a significant increase in the weight of the adductor longus muscle due to an increase in the number of fibers per unit cross-sectional area, though the mean size of both fast-twitch oxidative glycolytic and slow-twitch oxidative fibers was smaller in weight-lifting rats compared to controls. Light and electron microscopic examination showed that most weight-lifting rats exhibited longitudinally ‘split’ muscle fibers, suggesting a physiological adaptation to the stress of exercise [29]. Another study by Tamaki et al. [30] used a new training apparatus designed to mimic human squats, showing that squat training led to a significant increase in fiber branching and muscle hypertrophy due to hyperplasia [30].

In cats, weight-lifting exercises similarly led to muscle fiber branching. Gonyea et al. [31] investigated muscle hypertrophy induced by exercise and found that trained cats exhibited a significant increase in the number of muscle fibers in the flexor carpi radialis muscle due to fiber branching. This study provided evidence that exercise-induced hypertrophy involves not only an increase in cross-sectional area but also an increase in fiber number [31]. Further research by the same group in 1986 supported these findings, showing a significant increase in muscle weight and fiber number in the trained limbs of cats, reinforcing the role of prolonged weight-lifting exercise in inducing fiber branching in healthy muscle [32].

These findings indicate that muscle fiber branching is a common adaptive response to resistance training across different animal models, providing insights into muscle adaptation and regeneration that may be translatable to human exercise regimens.

Building on the findings from animal models, similar phenomena have been observed in humans undergoing extreme overload. A study of elite powerlifters by Eriksson et al. [18] found that these athletes exhibited muscle fiber branching (mid muscle), suggesting that extreme mechanical loading can result in abnormal muscle regeneration. The study observed that branched fibers varied in profile and some demonstrated recent degeneration or regeneration, likely due to continuous high mechanical stress on the muscles [18]. Beyond resistance-trained cohorts, team-sport and endurance athletes also experience repeated eccentric loading and transient muscle damage recovery cycles, providing a physiological context in which regenerative remodeling (and, potentially, imperfect fusion events) may occur, although branched fibers are rarely quantified directly in these studies. Conversely, immobilization/disuse primarily drives atrophy and altered remodeling, but the subsequent reloading phase can provoke secondary damage and regeneration, offering a useful comparator to injury/overload paradigms even though direct evidence linking disuse-reloading to branched fiber formation remains limited.

### 2.2. Distinctions and Misinterpretations in Hypertrophy and Hyperplasia

While fiber branching is often observed in conditions of mechanical overload, it is important to distinguish this phenomenon from classical hypertrophy (increased fiber size) and hyperplasia (increased fiber number). Several cross-sectional studies have reported apparent increases in muscle fiber number following overload, leading to suggestions of hyperplasia. However, work by Faber et al. [33] and others cautions that these findings may reflect the presence of longitudinally branched fibers rather than true de novo fiber formation. Importantly, studies relying solely on histological cross-sections are prone to overestimating fiber number in the presence of branching [34], whereas single-fiber isolation or 3D imaging provides a clearer picture of actual fiber architecture. This distinction is crucial for interpreting the adaptive significance of fiber branching, which may redistribute mechanical load across new branches while preserving total fiber count.

### 2.3. Synergistic Ablation and Electrical Stimulation-Induced Injury

Synergist ablation refers to a surgical overload technique that has been employed in rodents for decades to study skeletal muscle adaptations [35]. This method involves removing a primary muscle to overload a secondary synergistic muscle, causing significant hypertrophy. Studies have shown that synergist ablation leads to increased muscle fiber number and fiber branching [36].

**Figure 3:**
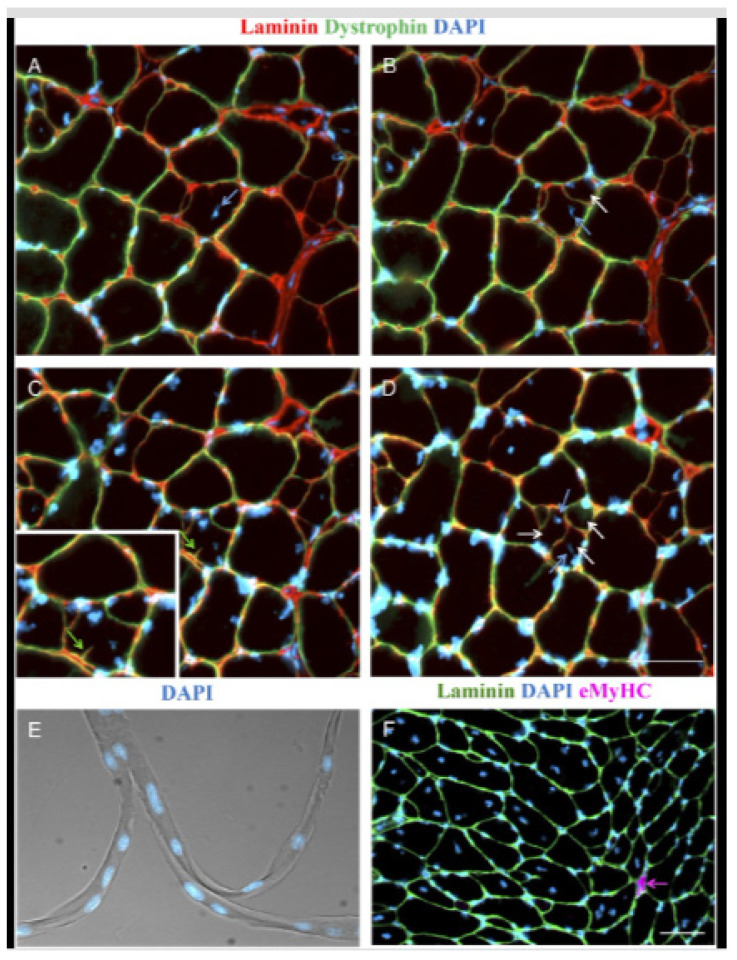
The appearance of split muscle fibers following 14 days of modified synergist ablation overload of the mouse plantaris, Murach et al. [8]. (**A**–**D**) illustrate fiber branching over ~50 μm on serial cross sections of a frozen plantaris muscle. Representative images show laminin and dystrophin to identify muscle fiber borders, and myonuclei. Dark arrows point to central myonuclei that appear prior to the appearance of each new branch in the muscle fiber (light arrows). (**E**) shows a phase-contrast image of a trifurcated single muscle fiber from this same mouse, along with myonuclei. (**F**) illustrates the extent of fiber branching morphology in this mouse, with minimal eMyHC expression (a marker of regeneration).

For example, Murach et al. [8] mechanically overloaded the plantaris of the mouse hindlimb by removing a portion of the gastrocnemius muscle whilst leaving the soleus intact. After 14 days, they observed significant plantaris hypertrophy characterized by an abundance of large muscle fibers with centrally located nuclei, minimal developmental myosin expression (<1% compared to ~30% in traditional synergist ablation) [37], and distinct muscle fiber branching, as shown in the stained isolated single-fibers (Figure 3). Serial cross-sections revealed that the emergence of centrally positioned nuclei preceded fiber branching, with continuous dystrophin and laminin present along the sarcolemma and basal lamina of each newly formed branch. This phenomenon supports the idea that fiber branching occurs as a consequence of intense muscle loading and independent of pathology [8]. However, due to the presence of satellite cells, it is possible that the mechanism of hypertrophic related fiber branching includes a myogenic response dependent on satellite fusion.

Electrical stimulation is a widely used technique to induce muscle contraction and can lead to muscle fiber injury and subsequent regeneration. In humans, electrical stimulation-induced injury has been shown to result in the occurrence of muscle fiber branching in regenerating muscle.

**Figure 4:**
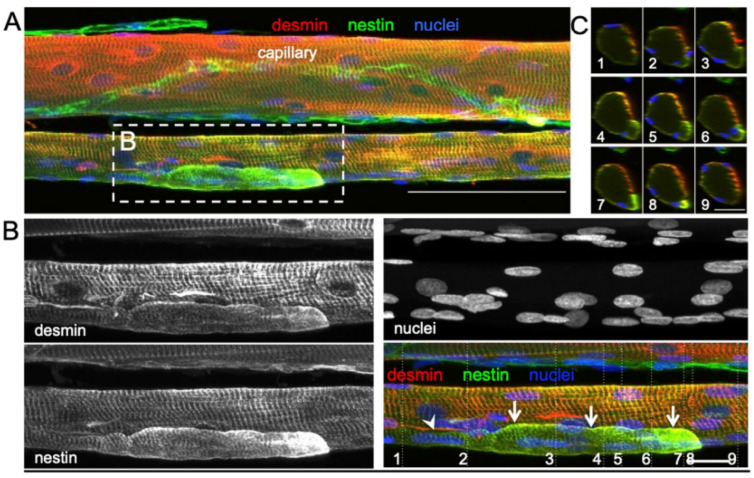
Confocal microscope images of three regenerating healthy human muscle fibers, 30 days post injury, Hojfeldt et al. [38]. (**A**,**B**) are stained for desmin (red), nestin (green), and nuclei (blue). (**A**) Maximum intensity projection of a 14-slice z-stack (100-μm scale bar), displaying a nestin + branch attached to a regenerating myofiber (the lower myofiber displayed here alongside an (upper) uninjured myofiber). White dotted square (**B**) refers to region containing a branched fiber. (**B**) Maximum intensity projection of a 25-slice z-stack (20-μm scale bar). Note the striated and nestin + segment (arrows) tightly associated with the parent myofiber. This branch displays a gradual increase in nestin immunoreactivity from the point of branching (at slice 1) towards its end (slice 9) shown on orthogonal slices (**C**).

Hojfeldt et al. [38] conducted a study of the vastus lateralis muscle of young healthy males exposed to electrical stimulation to systematically examine the process of muscle fiber regeneration and the occurrence of fiber branching in human skeletal muscle following injury. Muscle tissue samples collected 30 days after stimulation showed regular branching of small myofiber segments with a median branch length of 144 μm, often fused with the parent myofiber further along its length. Most of these branches were observed to fuse back with the parent fiber post-injury, with centralized myonuclei at branch points indicating a process of regeneration rather than splitting. This fusion process was associated with positive staining for nestin and neonatal myosin, indicating that the branches had an immature profile and were at an earlier stage of regeneration than the parent fibers (Figure 4). The branching observed in myofibers post-injury was attributed to incomplete regeneration, where myotubes fuse with existing damaged fibers rather than splitting apart.

### 2.4. Cardiotoxin- and Myotoxin-Induced Injury in Healthy Muscle

Muscle regeneration following cardiotoxin- and myotoxin-induced injury often results in significant muscle fiber branching. These toxins cause severe muscle damage, leading to a robust regenerative response characterized by muscle fiber branching. Although myotoxin-induced injury models are performed in otherwise healthy muscle, the acute damage and regenerative environment produced place them in an intermediate category (not fully pathological), but distinct from uninjured healthy muscle due to the transient regenerative stress.

Several different chemical agents are used to model acute muscle injury, each with distinct mechanisms and regenerative timelines. Barium chloride induces injury through ionic disruption and myofiber depolarization, leading to widespread necrosis. Notexin, a phospholipase A_2_ derived from Australian tiger snake venom, causes rapid and complete myofiber degeneration via membrane hydrolysis, while preserving the basal lamina and satellite cell niche [39]. In contrast, bupivacaine, a cardiotoxic local anesthetic, damages intracellular structures such as T-tubules and the sarcoplasmic reticulum. While all three agents elicit robust regeneration in otherwise healthy muscle, the extent and time course of degeneration–regeneration differ, with Notexin typically producing near-complete necrosis and recovery within 21 days [40], and bupivacaine or barium chloride leading to more variable damage and potentially longer-term structural changes [25,39,41].

In a study involving barium chloride-induced trauma to the gastrocnemius muscles of mice, Griffin et al. [42] analyzed serial transverse sections and found that 20–40% of muscle fibers exhibited branching just five days after treatment. This early appearance of branched fibers underscores the significant impact of chemical injury on muscle architecture and highlights the initial stages of fiber branch development. Notably, fiber branching is predominantly associated with regenerating muscle fibers, with minimal branching observed in non-regenerating fibers after chemical trauma induced by agents like cardiotoxins or barium chloride [4]. A substantial proportion of regenerating fibers (25–60%) display branching within 0 to 21 days post-injury, and this remains the case even after extended recovery periods of up to six months. These findings suggest that chemical trauma has prolonged effects on muscle regeneration, leading to persistent fiber branching over time.

Notexin produces clearly defined phases of degeneration and regeneration when applied to rodent hind limb muscles [25,39,41]. Notexin is favored in muscle regeneration studies due to its greater myotoxic damage and slower functional repair in skeletal mouse studies compared to other myotoxins like bupivacaine and barium chloride [43]. When injected into the extensor digitorum longus (EDL) of wild-type mice, Notexin leads to complete fiber necrosis and loss of functional capacity after three days, in contrast to the 45% breakdown observed with other myotoxins [39].

In wild-type mice, a single Notexin injection to the EDL results in regenerated muscle containing branched fibers by 21 days post-injection [40], the muscle had functionally recovered, and was able to produce pre-injury levels of absolute maximal force with similar levels of passive and active stiffness. The twitch kinetics had also recovered to pre-injury levels. The regenerated muscles were mechanically stable and showed the same levels of eccentric force loss as controls. Approximately 93% of the fibers were centrally nucleated, and 29% were branched [40] suggesting that in otherwise healthy muscle, this level of structural differences have no deleterious impact on function.

Previous studies in rats have demonstrated that multiple rounds of muscle injury can result in the formation of ‘complex’ branched fibers with multiple branches per muscle fiber. An early study lead by Sadeh et al. [17] in rat tibialis anterior muscle reported extensive fiber branching after six months of weekly bupivacaine-induced muscle damage followed by a two-month recovery period. They found marked differences in fiber size variability, centralized nuclei, extensive fiber branching, and whorled fibers. Similarly, Tamaki and Akatsuka [44] conducted a study involving ten rounds of cardiotoxic (bupivacaine) insult to the plantaris muscle in healthy rats. Although they could not quantify the number of branched fibers using cross-section analysis, they reported that their serial injury protocol produced complex branched fibers after mechanical isolation, with up to ten branches per fiber. Further investigations by Tamaki et al. [24] examined the effects of numerous complex branched fibers on whole muscle contractile properties. In the same model, researchers found that repeated bupivacaine injury resulted in an 8-fold increase in the number of branched fibers in the plantaris muscles with about 70% of the fibers containing complex branching. These complex branches contained ten or more muscle fibers fused together with many thin, thick, long and short daughter branches. Evaluating plantaris muscle function identified the time to peak tension of twitch and tetanus, as well as half relaxation time, were significantly longer in treated muscles compared to controls, possibly reflecting shorter stiffer branched fibers or potential disorganization of myofibrils and force vectors discussed later in this review [45].

The occurrence of muscle fiber branching under various conditions, such as extreme mechanical loading, synergistic ablation, electrical stimulation-induced injury, and myotoxin exposure, suggests that this phenomenon may serve as a broader adaptive response rather than a merely pathological event. In scenarios of extreme mechanical loading, such as resistance training or synergistic ablation, the splitting and branching of muscle fibers appear to be a means by which the muscle redistributes the stress across a greater number of smaller fibers. This redistribution likely reduces the mechanical strain on any single fiber, thereby preventing potential damage that could arise from excessive load bearing. The increased fiber number, as seen in studies involving bird wing-loading models and weight-lifting rodents, supports the notion that fiber branching could contribute to an overall hypertrophic response [33]. This hypertrophy, in turn, may help to stabilize the muscle structure and maintain function under increased physical demands.

The slowing of contractile properties observed in muscles with a high degree of fiber branching, such as those subjected to repeated chemical injury [17,44], further supports the idea that this branching could serve a protective role. Slower contractile kinetics, as evidenced by prolonged time to peak tension and half-relaxation times in branched fibers, may reduce the likelihood of rapid, high-force contractions that would exacerbate muscle damage [24]. This shift towards slower, more controlled muscle contractions might enhance the muscle’s resilience to repeated bouts of mechanical stress, thereby preserving muscle integrity and function over time. The presence of branched fibers in regenerating muscles following injury, particularly in models where central nuclei and immature myofibers are observed, suggests that branching may also be a part of the muscle’s natural repair process [4]. By generating new branches from existing fibers, the muscle may be attempting to restore or even augment its functional capacity in response to damage.

Despite the potential protective role of fiber branching in response to overload and injury in healthy muscles, its occurrence in muscular dystrophies like Duchenne muscular dystrophy presents a paradox. In this disorder, fiber branching, rather than being a beneficial adaptation, contributes to the progressive deterioration of muscle function.

## 3. Muscle Fiber Branching in Duchenne Muscular Dystrophy

In humans, the history of branched fibers in disease date back to reports from Erb in 1891 and Krosing in 1892, where branching was first reported in boys with Duchenne muscular dystrophy (DMD), associated with deficiency of the sarcolemmal protein dystrophin. As a condition that involves chronic degeneration and regeneration of muscle fibers, it is perhaps not surprising to see branched fibers in muscle biopsies from boys with DMD [6,10,46].

**Figure 5:**
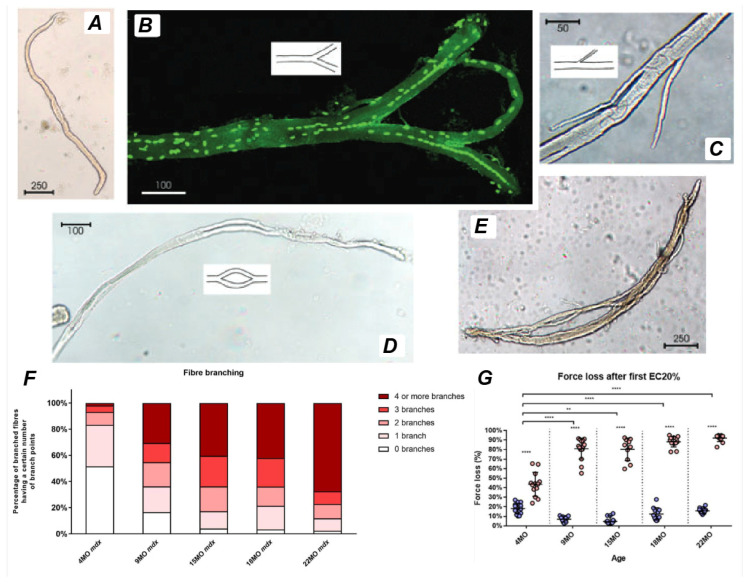
Examples of branched fibers. Low powered images of enzyme dispersed single muscle fibers from EDL (Extensor digitorum longus) muscles of mdx mice from Chan and Head [5]. (**A**) Morphologically normal unbranched fiber. (**B**) Branched fiber with a bifurcation. (**C**) A fiber with two small branches. (**D**) A fiber that branches and recombines. (**E**) Fibers with highly complex branching patterns. (**F**) The extent of branched fiber morphology in the mdx EDL muscle was quantified through enzymatic digestion across different age groups between 4 months and 22 months. Fibers exhibiting complex branching (defined as having four or more branches per fiber) are expressed as a percentage of the total fiber count. (**G**) Additionally, the loss of force following a single eccentric contraction at 10% strain is presented as a percentage of the initial force. (**F**,**G**) is derived from Kiriaev et al. [54]. For interleaved scatterplots, the horizontal line indicates mean ±SD. Statistical differences between age groups are displayed above the graph and differences displayed within the graphs are differences between genotypes assessed by two-way ANOVA, post hoc analysis using Sidak’s multiple comparisons test. **** *p* < 0.0001 and ** 0.001 < *p* < 0.01.

Branched fibers are prominently observed in the *mdx* mouse, a widely used animal model for DMD, within various hindlimb muscles (Figure 5) [4,21,33,45,47,48,49,50], as well as in the diaphragm [51]. The abrupt onset of myonecrosis at 21 days stimulates robust muscle fiber regeneration and the occurrence of branched fibers. Morphological fiber branching increases in prevalence as the animal ages, as first demonstrated by Chan et al. [47] in fast-twitch EDL muscles of the *mdx* mouse where less than 20% of fibers are branched two months of age, as opposed to the 90% when the animal is six months old.

Regenerated *mdx* muscle fibers contain centrally aligned nuclei and regularly show a range of structural malformations from small, single branches to complex branching of fibers (Figure 5). It is possible that the branching itself maintains the nuclei in their central position in the regenerated *mdx* muscle fibers. Depending on the model of injury, necrosis can occur segmentally, affecting only part of the myofiber and can occur simultaneously at multiple sites along the length of the muscle fiber rather than the beginning or end [52,53]. Thus, incomplete fusion of myotubes at these locations can explain the variation in branching morphology ranging from simple bifurcations (Figure 5B) to complex intertwining syncytia (Figure 5E).

Recently, Carlson [55] proposed a novel hypothesis suggesting that the absence of dystrophin leads to unrestrained satellite cell activation and proliferation, independent of prior fiber injury. This perspective aligns with recent insights moving away from dystrophin’s role in membrane stability [56] challenging the long-held belief of dystrophin as primarily a membrane stabilizer. Supporting this regulatory role, myostatin inhibitors have been shown to exacerbate fiber branching in dystrophic muscles in *mdx* mice [57]. Unlike healthy postnatal muscle, where myostatin inhibition promotes fiber hypertrophy without inducing significant hyperplasia [58], dystrophic muscles treated with myostatin inhibitors exhibited no hypertrophy at the individual fiber level, but showed apparent hyperplasia in cross-section, likely reflecting an increase in branched fibers rather than true fiber number. This response underscores the critical role of dystrophin in satellite cell regulation, with its absence driving continuous activation and proliferation that accelerates the formation of branched and fragile, progressively weakening dystrophic muscles.

It is also important to note that branched fibers, although much less common, can occur in non-dystrophic, non-injured muscles in both humans [13,59] and mice [4]. For example, studies have observed that control mice possess a small percentage of branched fibers, typically comprising approximately 1% of their total muscle fiber population [54]. Interestingly, when both control and *mdx* mice were subjected to mild eccentric contractions (5–10% eccentric strain), younger *mdx* mice exhibited a low force deficit similar to that of the controls, whereas older *mdx* mice showed a significantly greater force loss [47,60].

While branched fibers are present in control muscles, their prevalence and impact become more pronounced in dystrophic conditions, particularly with advancing age or repeated stress. This highlights the need for further investigation into the relationship between the extent of fiber branching and various aspects of muscle function, including susceptibility to damage from eccentric contractions. Understanding how fiber branching affects muscle mechanics, force transmission, and overall muscle integrity could provide deeper insights into both adaptive and maladaptive responses in muscle tissue. This is particularly important in conditions such as muscular dystrophies, where the presence of branched fibers may represent an initial compensatory adaptation that later becomes detrimental.

## 4. Consequences of Muscle Fiber Branching

### 4.1. Branched Fibers Are Shorter and Stiffer as a Compensatory Adaptation

Branched fibers may serve as a compensatory adaptation to mechanical stress, but their accumulation fragments the muscle into shorter, irregular fiber segments that increase stiffness. This phenomenon is evident in *mdx* mice [60], where an age-linked rise in muscle stiffness correlates with the appearance of branched fibers. The resulting increase in passive stiffness, which becomes more pronounced with age, is hypothesized to arise from the mechanical properties of these fragmented segments, including altered myofibrillar alignment and impaired elastic recoil, contributing to the overall structural rigidity of the muscle. While historical accounts of branched muscle fibers date back over 100 years, the physiological implications of branching in regenerated muscle remain uncertain [12,61]. One hypothesis is that fiber branching may initially provide a protective function, with regenerated branches aligning parallel to existing fibers along the muscle’s long axis, effectively increasing stiffness and cross-sectional area [33].

The complex arrangement of structures within whole muscle enables force generation and transmission in myofibrils along the length of the fiber to a well-developed connective tissue matrix [62]. However, muscle fibers do not run from tendon plate to tendon plate and fibers within a motor unit may not extend the entire fascicle length but contain multiple units with origin and insertion points in the center of the muscle belly [63,64]. While whole muscle length remains constant from adolescence to senescence in dystrophic animals [21,47,54,60,65,66], the accumulation of fiber branching with age suggests that some fibers may fail to span the full muscle length. Instead, they may terminate within the muscle belly, forming mechanically distinct segments. This is analogous to interfascicular terminating fibers which transmit tension via fiber to fiber or collagenous junction [67]. Such internal terminations may contribute to stiffness and force transmission defects in dystrophic muscles. Multiple studies performed by Karpati et al. [68] suggest that regenerated fibers never reach the expected cross sectional area and remain around 80% the size of normal fibers, even at maximal growth. This size deficit is distinct from fiber branching and may represent an adaptive mechanism to reduce stress within vulnerable dystrophic fibers.

Studies performed by Petrof et al. [69] & Petrof [70] on dystrophic EDL and diaphragm muscle fibers suggest that shear stress across muscle membranes is the main predictor of injury. This is based on the observation that larger muscle fibers, which have a lower surface area-to-volume ratio compared to smaller fibers, are more easily damaged. The authors proposed that the shear stress generated by the myofibrils within these larger fibers is responsible for the muscle damage. In dystrophic muscle, increased stiffness may instead reflect changes in sarcomeric properties, titin isoforms, or extracellular matrix organization. Functionally, higher passive stiffness may enhance stability against small positional perturbations, serving as a compensatory mechanism in the face of impaired contractile force. Likewise, reduced range of motion may reflect an adaptive shift towards passive tension reliance [71].

### 4.2. Hypertrophy and Low Specific Force in Mdx Muscles

Recent studies have explored the progression of muscle pathology in *mdx* mice over their lifespan to better understand how this model mimics DMD [66]. Key pathological features, such as endplate fragmentation, centrally located nuclei, and fiber branching increase progressively during the first year of life in *mdx* mice. Notably, while individual *mdx* muscle fibers exhibit hypertrophy at the myofiber level, there is no corresponding increase in whole muscle hypertrophy. This finding contrasts with earlier studies reporting significant whole muscle hypertrophy of 20–30% in the *mdx* EDL and 20–60% in the tibialis anterior [33,47,48,65,72,73,74,75,76,77,78,79,80].

The observed hypertrophy at the fiber level is often assumed to result from an increase in fiber number; however, it may also be attributable to fiber branching. This is particularly difficult to distinguish histologically, as branched fibers can resemble increased fiber numbers when viewed in cross-sections [4,33,81]. Despite the hypertrophy of individual fibers, there is a well-documented decrease in specific force in dystrophic EDL muscles and myofibrils as the animal ages. This decline in force correlates with the increasing severity of repeated cycles of degeneration and regeneration observed in the *mdx* mouse model [47,65,76,77,82,83,84,85,86].

**Figure 6:**
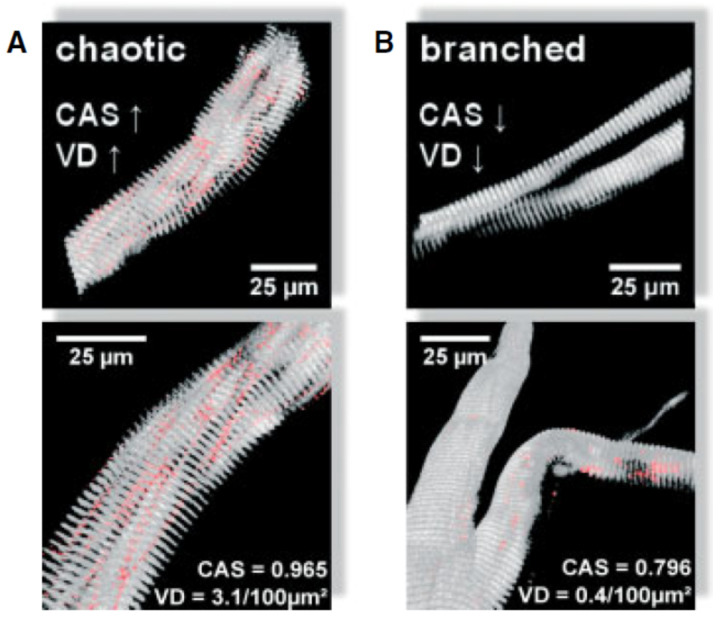
Chaotic and branched micro-architectural phenotypes Buttgereit et al. [45]. The chaotic type (**A**) is characterized by a high vernier density (red, VD) and cosine angle sum (calculated force vector, CAS). (**B**) Branched fibers include bundles of myofibrils separated but continuous with the main trunk, showed minimal alteration resulting in low vernier density and cosine angle sums.

Figure 6 illustrates different micro-architectural phenotypes, including chaotic and branched fibers [45]. Repeated cycles of degeneration and regeneration result in structural remodeling of branched myofibrils, which often appear hypercontracted, tilted, twisted, and deviated when observed using second harmonic generation (SHG) microscopy [87]. These morphological alterations, described as ‘verniers’ by Friedrich’s group, affect neighboring sarcomere activation and lead to unsynchronized muscle contraction, contributing to an estimated 20% reduction in force production. The frequency of these misalignments, or verniers, increases with age, further contributing to the decline in specific force in dystrophic muscle [45]. Interestingly, younger regenerated fibers without branches have shown misaligned contractile architectures termed ‘chaotic fibers’, which are intermediate between normal and branched fibers and are predicted to contribute to approximately 50% of the progressive force loss with age. As dystrophic animals age, the increased degree of fiber branching likely results in more extensive structural remodeling and a higher occurrence of verniers, further exacerbating the reduction in muscle force. This structural complexity not only compromises the muscle’s ability to generate force but also affects its resilience to mechanical stress. The abnormal branching and misalignment within the muscle fibers may create points of weakness that are particularly vulnerable to damage during eccentric contractions, where muscles are lengthened under tension.

Understanding how fiber branching, myofibril misalignment and higher occurrence of verniers contributes independently or together to predispose these vulnerabilities is essential, as it may explain the heightened susceptibility of dystrophic muscles to eccentric-induced damage. In the following section, we delve deeper into the role of fiber branching in influencing muscle response to eccentric contractions, exploring how these architectural changes might predispose muscle fibers to injury and affect overall muscle function.

### 4.3. Does Fiber Branching Contribute to the Susceptibility of Dystrophic Muscles to Eccentric-Induced Damage?

The role of fiber branching in contributing to the susceptibility of dystrophic muscles to eccentric-induced damage is increasingly recognized in muscular dystrophy research. In patients with DMD, extensive fiber branching has been associated with reduced mobility [10]. Several studies suggest that branch points within muscle fibers are particularly vulnerable to contractile injury. The unique architecture of these branched fibers, characterized by irregular diameters and cross-sectional areas, creates sites where high shear stress may lead to fiber rupture during intense contractile activities [88].

During the progression of DMD, the cyclic process of muscle degeneration and regeneration produces fibers with varying diameters and branching patterns, contributing to structural instability. This morphological diversity results in differences in action potential propagation velocities along the fiber, with smaller-diameter branches exhibiting delayed transmission compared to larger branches. According to ‘cable theory’, electrical propagation is more likely to fail in smaller branches, potentially disrupting the coordinated activation of muscle fibers [89,90,91]. Computational models have further demonstrated that branched fibers exhibit non-uniform strain distributions, particularly at branch points, increasing the likelihood of stretch-induced damage [92]. This non-uniformity becomes more pronounced as the model moves from smaller to larger branches, highlighting the mechanical disadvantages posed by fiber branching.

Further studies support the idea that fiber branching affects [Ca^2+^] handling within dystrophic muscles. Smaller-diameter branches at branch points display faster [Ca^2+^] uptake kinetics into the sarcoplasmic reticulum (SR), reflecting a more rapid response to contraction but also a heightened risk of asynchronous activation [93]. These findings suggest that the irregular structure of branched fibers contributes to mechanical instability and a higher propensity for rupture at branch nodes due to delayed and uncoordinated electrical activation.

Studies of the *mdx* mouse model have provided additional insights into the differential properties of branched versus unbranched fibers. For instance, Lovering et al. [49] demonstrated that branched fibers in *mdx* mice show reduced SR Ca^2+^ release and clearance when exposed to osmotic stress, indicating that these branches are more susceptible to stress-induced dysfunction. This vulnerability is compounded by the altered action potential characteristics observed at branch points, including increased time to peak and broader action potentials compared to non-branched segments [94]. These electrical and calcium signaling abnormalities may underlie the decreased force generation capacity and increased damage susceptibility observed in dystrophic muscles.

Experimental data also reveal that branched fibers in dystrophin-deficient muscles are structurally weaker. Head [88] showed that branched muscle fibers from *mdx* mice were more likely to break under maximal Ca^2+^ activation compared to unbranched fibers, suggesting a direct impact of branching on structural integrity. Moreover, increased mechanical stress at branched regions has been linked to elevated production of reactive oxygen species and enhanced Ca^2+^ influx through mechanosensitive channels, leading to localized membrane damage at branch points [56]. These findings imply that branch points in dystrophic muscles not only increase susceptibility to acute mechanical tears, but may also serve as focal points for secondary damage pathways involving pathological Ca^2+^ handling.

Figure 5F,G illustrates the correlation between complex branching and force loss in the EDL muscle of *mdx* mice from 4 to 22 months of age. Studies using enzymatic digestion across different age groups have quantified branched fiber morphology, revealing that fibers with complex branching (defined as four or more branches per fiber) are more prevalent in aged muscles. Furthermore, the force loss following a single eccentric contraction at 10% strain is significantly higher in muscles with extensive branching, underscoring the link between branching complexity and mechanical vulnerability.

The relationship between fiber branching and susceptibility to eccentric-induced damage is particularly evident in older *mdx* mice. As these mice approach the end stage of dystrophinopathy, their muscles consist almost entirely of complex branched fibers, leading to a dramatic and abrupt loss of force (~65%) following the first eccentric contraction [54,60]. This pattern contrasts sharply with younger *mdx* mice, where eccentric contraction-induced damage is characterized by a more gradual, incremental loss of force [95,96,97,98]. While low levels of fiber branching may initially provide a protective adaptation against muscle damage [33], excessive and complex branching eventually becomes maladaptive, leading to increased mechanical weakness and heightened susceptibility to injury [45,87].

Collectively, these findings support a two-phase model of muscle damage in DMD, where fiber branching initially serves a compensatory function but becomes detrimental as branching complexity increases. This model underscores the need for therapeutic strategies targeting early intervention before the transition from adaptive to maladaptive branching occurs, which could potentially mitigate the progression of muscle weakness and susceptibility to damage in dystrophic muscles.

### 4.4. A Model to Link Fiber Branching and Progressive Dysfunction in Dystrophic Mice Muscle

**Figure 7:**
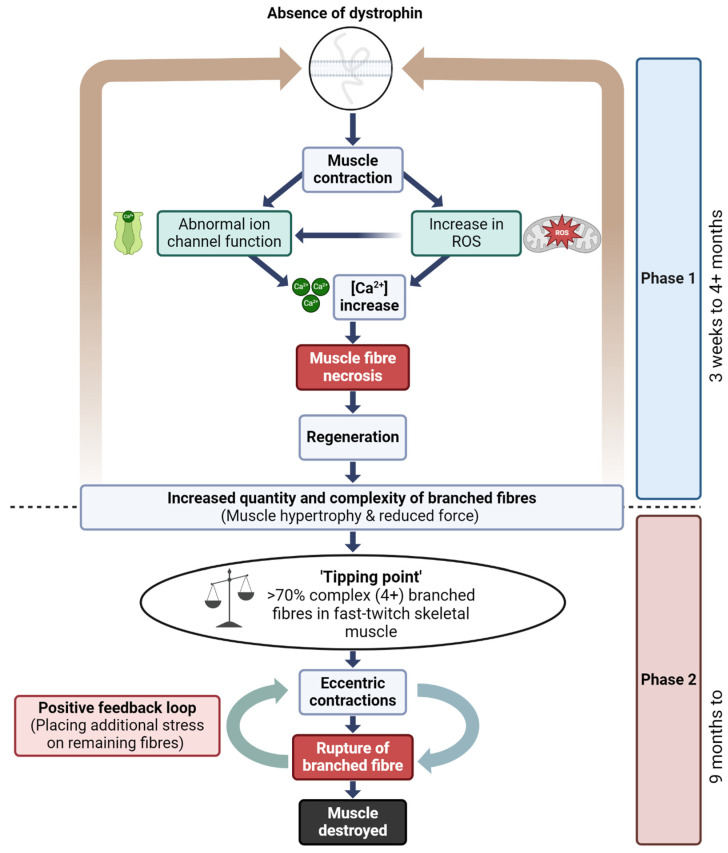
Two-phase model of disease pathogenesis in *mdx* mice, Kiriaev et al. [54]. This model illustrates the progression of muscle pathology in fast-twitch muscles of mdx mice in two phases. **Phase one** is characterized by the absence of dystrophin, leading to repeated cycles of muscle fiber necrosis and regeneration due to elevated intracellular calcium levels [Ca^2+^]^in^, increased reactive oxygen species (ROS), and abnormal ion channel activity. These cycles result in the formation of abnormally branched dystrophin-deficient muscle fibers, which increase in complexity over time. **Phase two** is triggered when fiber branching reaches a critical threshold, or ‘tipping point’, resulting in fiber rupture during mechanical stress, especially eccentric contractions. This phase involves a positive feedback loop of progressive muscle damage as broken fibers fail to support muscle contraction, leading to further strain on remaining fibers and eventual widespread muscle degeneration. Depending on mechanical stress, both phases can occur concurrently.

The pathophysiology of DMD in skeletal muscle has been conceptualized in our laboratory as a two-phase process (Figure 7) that helps contextualize the structural consequences of repeated injury in dystrophin-deficient muscle [5,12,47,54,60,88]. While not mutually exclusive, these phases delineate an early period of compensatory regeneration and a later period characterized by increased structural instability due to cumulative fiber branching.

However, it is important to emphasize that fiber branching is unlikely to be the sole driver of disease progression. Multiple studies have shown that limb muscles in mdx mice do not undergo the same extent of mass loss or fibrosis observed in DMD patients, and in fact may preserve muscle mass with age [65]. Additionally, muscle regeneration in DMD patients remains relatively constant across age, as shown by stable embryonic myosin expression [99], and muscle mass measurements in DMD patients show no sharp decline during adolescence [100]. The diaphragm of mdx mice does undergo progressive fibrosis [101], suggesting that disease severity may be muscle specific.

Thus, while the two-phase model provides a structural framework for understanding how fiber branching may contribute to mechanical failure in dystrophic muscle, we recognize that disease progression in DMD involves a complex interplay of factors. These include inflammation, fibrosis, metabolic remodeling, and compensatory adaptations, all of which can vary by muscle group and age. The proposed model is therefore intended as a conceptual scaffold rather than a deterministic sequence and should be interpreted alongside these broader pathological contributors.

### 4.5. Reversible and Irreversible Recovery of Force Post Eccentric Contraction

Dystrophin is a critical protein involved in several key signaling pathways in skeletal muscle, including those regulating nitric oxide (NO) production, modulation of stretch-sensitive ion channels, the entry of Ca^2+^ and Na^+^ ions [102,103] and controlling the production of ROS [104] (Figure 7). In the absence of dystrophin, these signaling pathways are disrupted, leading to pathological influxes of intracellular calcium [Ca^2+^]^in^, which trigger skeletal muscle fiber necrosis [56]. This disruption is central to the two-phase model of DMD pathogenesis, where the absence of dystrophin initiates a cycle of muscle fiber degeneration and regeneration, resulting in the formation of branched fibers. The complexity and number of these branched fibers increase with each regenerative cycle, particularly as the dystrophic muscle ages, ultimately leading to catastrophic membrane rupture during both isometric and eccentric contractions.

Empirical evidence supports this model, illustrating the differential recovery capacities of dystrophic muscle following eccentric contractions. For instance, Olthoff et al. [105] demonstrated that young (3-month-old) *mdx* mice experience a 90% loss of force in the EDL muscle after 10 eccentric contractions but can recover up to 65% of force within 120 min. Notably, when eccentric contractions were spaced 30 min apart, there was no irreversible loss of force, suggesting that transient, redox-based inhibition of contractility could protect dystrophin-deficient muscles from catastrophic structural damage during subsequent contractions. A similar study by Lindsay et al. [85] found that young *mdx* mice exhibited a 75% force loss following eccentric contractions, yet recovered up to 64% of their original force within 60 min post-contraction. These findings indicate a significant reversible component of force loss in younger muscles that have not yet reached the ‘tipping point’ of extensive branching complexity.

Several studies have also explored the variability in force recovery among young *mdx* mice, showing a range of outcomes depending on muscle condition and age [82,84,106,107]. Interestingly, the administration of the macrophage-synthesized antioxidant 7,8-Dihydroneopterin to young *mdx* muscles improved protection against eccentric contractions and enhanced force recovery to 81% [85]. These results suggest that the restoration of isometric tetanic force through antioxidant treatment may involve reversible oxidation of proteins that regulate muscle contraction.

However, the capacity for force recovery diminishes significantly with age and the degree of fiber branching. Studies have shown that while young dystrophic muscles can recover a substantial portion of force after eccentric contractions, aged and senescent *mdx* mice only recover about 20–30% of their starting force following a 120 min recovery period [54]. This reduced recovery is attributed to the presence of complex branched fibers, which are prone to rupture under mechanical stress. In contrast, younger *mdx* mice, which have fewer complex branches, exhibit less severe force loss. The variability in force recovery among *mdx* muscles can thus be explained by differences in the extent and complexity of fiber branching [82,84,85,105,106,107,108,109].

Clinically, understanding the dynamics of reversible versus irreversible force loss is crucial for identifying therapeutic windows in DMD patients. The observation of irreversible force loss due to acute membrane rupture in complex branched fibers suggests that interventions must occur before muscle branching reaches a critical ‘tipping point’. Preventing the initial pathological increase in Ca^2+^ could halt the disease process before the muscle enters the cyclic degeneration and regeneration phase that leads to mechanical compromise. For patients receiving treatments in the later stages of DMD, where muscle branching complexity is more advanced, therapeutic success rates will likely diminish. Therefore, early genetic rescue strategies and preclinical drug studies are essential before the onset of extensive pathological fiber branching that compromises muscle function.

## 5. Future Directions—Targeting Fiber Branching as a Therapeutic Intervention

Muscle fiber branching, while historically considered a downstream consequence of injury or disease, is increasingly recognized as a modifiable feature of muscle architecture with therapeutic potential. The strategies highlighted in this section were selected because they either directly modulate the formation of branched fibers or alter the regenerative processes known to influence branching frequency, morphology, or impact. These interventions represent emerging efforts to harness or suppress fiber branching depending on its adaptive or pathological context. Targeting the regulatory pathways that govern myotube fusion, fiber stability, and remodeling such as olfactory receptor 23 (mOR23) signaling, satellite cell control, or fiber-type shifting may allow us to reshape how muscle responds to injury and prevent the formation of structurally vulnerable branched fibers in conditions like DMD.

Grace Pavlath’s work on mOR23 has provided valuable insights into the regulation of muscle fiber branching [110]. By overexpressing mOR23, Pavlath’s group demonstrated a significant reduction in fiber branching in regenerating muscles, which translated to reduced contraction-induced membrane permeability (Evans blue dye uptake) in dystrophic *mdx* mice. This finding raises the possibility of targeting mOR23 or similar molecules to reduce fiber branching and protect against mechanical stress in dystrophic muscles.

In addition to molecular targets like mOR23, targeting satellite cell activity may provide novel therapeutic avenues for managing fiber branching in DMD. Carlson [55] highlighted that treatments preventing unrestrained satellite cell activation could mitigate the formation of structurally weak branched fibers. Myogenic regulatory factors (MRFs) and insulin-like growth factors (IGFs) play crucial roles in muscle regeneration by regulating satellite cell activation, proliferation, and fusion [111]. Enhancing these processes could potentially improve the efficiency of myotube fusion and reduce the incidence of branched fibers. Moreover, neuromuscular electrical stimulation has been shown to enhance satellite cell fusion with mature myofibers, which may further decrease the formation of branched fibers by promoting more complete and robust fusion during muscle regeneration [112].

Another promising strategy involves driving a shift toward a slower, more protective muscle phenotype. Selsby et al. [113] first demonstrated that transgenic overexpression of PGC1α in the *mdx* mouse increased oxidative fiber content (a 3-fold increase in type 1 myosin heavy chain), enhanced resistance to contraction induced injury, and improved specific force production by 12%. More recently, a pharmacological approach using the fast myosin inhibitor EDG-5506 achieved similar protective outcomes by selectively inhibiting force generation in fast skeletal fibers while sparing slow and cardiac muscle [114]. EDG-5506 significantly reduced membrane injury, force loss, and fibrosis in both *mdx* and canine DMD models. These findings support fast fiber specific targeting as a viable strategy to reduce mechanical stress in dystrophic muscle. Notably, slow oxidative muscles such as the soleus are naturally enriched in utrophin, with expression ~2–3 fold higher than in fast EDL muscle of *mdx* mice [115], offering a potential mechanistic explanation for their resistance to damage and enhanced regenerative potential [116].

## 6. Conclusions and Perspectives

Muscle fiber branching is a complex phenomenon that serves dual roles in muscle physiology: as a protective adaptation in healthy muscle and as a detrimental feature in degenerative muscle diseases like DMD. In the context of healthy muscle, fiber branching can help redistribute mechanical stress, promote hypertrophy, and serve as a defense mechanism against injury. Conversely, in dystrophic muscle, excessive and complex fiber branching leads to mechanical instability, increased susceptibility to damage, and a decline in muscle function. This tension, between adaptation and pathology, is intentionally left unresolved, as it reflects the current state of the field and highlights a key opportunity for future investigation.

The dichotomy of muscle fiber branching in health and disease underscores its context-dependent nature. In healthy muscle, branching may be part of an adaptive response, helping to maintain muscle integrity under conditions of extreme mechanical load or injury. However, in DMD, the absence of dystrophin triggers a pathological cascade where repeated cycles of degeneration and regeneration lead to the formation of complex branched fibers. These fibers, while initially compensatory, eventually become sites of structural weakness, contributing to progressive muscle degeneration and functional decline.

Understanding this dual role of fiber branching is crucial for developing targeted therapeutic strategies. The two-phase model of DMD pathogenesis presented in this review provides a framework for understanding how muscle pathology progresses from initial regenerative responses to terminal muscle degeneration. This model highlights the importance of early intervention to prevent fiber branching from reaching a level where it exacerbates muscle damage.

Future research should focus on modulating the processes that govern muscle fiber branching and fusion to harness their protective potential while minimizing their pathological consequences. Investigating molecular targets such as mOR23, MRFs, and IGFs, as well as promoting shifts toward slower muscle phenotypes, represents promising avenues for therapy. By better understanding the molecular mechanisms underlying fiber branching, we can move towards innovative therapeutic approaches that enhance muscle regeneration and improve outcomes for patients with muscle diseases.

In summary, muscle fiber branching is not inherently detrimental but becomes so under specific pathological conditions. A nuanced understanding of the balance between adaptation and pathology in muscle tissue will be essential for designing effective therapies for muscular dystrophies and other muscle-related diseases.

## Data Availability

No new data were created or analyzed in this study. Data sharing is not applicable to this article.

## Abbreviations

The following abbreviations are used in this manuscript:

DMD	Duchenne Muscular Dystrophy
*mdx*	Dystrophin-deficient mouse
EDL	Extensor Digitorum Longus
SR	Sarcoplasmic Reticulum
ROS	Reactive oxygen species
NO	Nitric oxide
[Ca^2+^]^in^	Intracellular calcium concentration
CD68	Cluster of differentiation 68 (macrophage marker)
eMyHC	Embryonic myosin heavy chain
SHG	Second harmonic generation microscopy
VD	Vernier density
CAS	Cosine angle sum
mOR23	Murine olfactory receptor 23
MRFs	Myogenic regulatory factors
IGFs	Insulin-like growth factors
PGC1a	Peroxisome proliferator-activated receptor gamma coactivator 1-alpha
EDG-5506	Small molecule fast skeletal myosin inhibitor

## References

[1] Ciciliot S., Schiaffino S. (2010). Regeneration of mammalian skeletal muscle. Basic mechanisms and clinical implications. Curr. Pharm. Des..

[2] Grounds M.D. (2014). The need to more precisely define aspects of skeletal muscle regeneration. Int. J. Biochem. Cell Biol..

[3] Ontell M., Hughes D., Bourke D. (1982). Secondary myogenesis of normal muscle produces abnormal myotubes. Anat. Rec..

[4] Pichavant C., Pavlath G.K. (2014). Incidence and severity of myofiber branching with regeneration and aging. Skelet. Muscle.

[5] Chan S., Head S.I. (2011). The role of branched fibres in the pathogenesis of Duchenne muscular dystrophy. Exp. Physiol..

[6] Schmalbruch H. (1984). Regenerated muscle fibers in Duchenne muscular dystrophy: A serial section study. Neurology.

[7] Dueweke J.J., Awan T.M., Mendias C.L. (2017). Regeneration of Skeletal Muscle After Eccentric Injury. J. Sport Rehab..

[8] Murach K.A., White S.H., Wen Y., Ho A., Dupont-Versteegden E.E., McCarthy J.J., Peterson C.A. (2017). Differential requirement for satellite cells during overload-induced muscle hypertrophy in growing versus mature mice. Skelet. Muscle.

[9] Martin R.A., Buckley K.H., Mankowski D.C., Riley B.M., Sidwell A.N., Douglas S.L., Worth R.G., Pizza F.X. (2020). Myogenic Cell Expression of Intercellular Adhesion Molecule-1 Contributes to Muscle Regeneration after Injury. Am. J. Pathol..

[10] Bell C.D., Conen P.E. (1968). Histopathological changes in Duchenne muscular dystrophy. J. Neurol. Sci..

[11] Ontell M., Feng K.C. (1981). The three-dimensional cytoarchitecture and pattern of motor innervation of branched striated myotubes. Anat. Rec..

[12] Head S.I., Hegde M. (2012). A Two Stage Model of Skeletal Muscle Necrosis in Muscular Dystrophy—The Role of Fiber Branching in the Terminal Stage. Muscular Dystrophy.

[13] Schmalbruch H. (1976). The morphology of regeneration of skeletal muscles in the rat. Tissue Cell.

[14] Mackey A.L., Kjaer M. (2017). The breaking and making of healthy adult human skeletal muscle in vivo. Skelet. Muscle.

[15] Kiriaev L., Baumann C.W., Lindsay A. (2023). Eccentric contraction-induced strength loss in dystrophin-deficient muscle: Preparations, protocols, and mechanisms. J. Gen. Physiol..

[16] Gutiérrez J.M., Núñez J., Díaz C., Cintra A.C., Homsi-Brandeburgo M.I., Giglio J.R. (1991). Skeletal muscle degeneration and regeneration after injection of bothropstoxin-II, a phospholipase A2 isolated from the venom of the snake Bothrops jararacussu. Exp. Mol. Pathol..

[17] Sadeh M., Czyewski K., Stern L.Z. (1985). Chronic myopathy induced by repeated bupivacaine injections. J. Neurol. Sci..

[18] Eriksson A., Lindström M., Carlsson L., Thornell L.-E. (2006). Hypertrophic muscle fibers with fissures in power-lifters; fiber splitting or defect regeneration?. Histochem. Cell Biol..

[19] Hall-Craggs E.C.B. (1972). The significance of longitudinal fibre division in skeletal muscle. J. Neurol. Sci..

[20] Swash M., Schwartz M.S. (1977). Implications of longitudinal muscle fibre splitting in neurogenic and myopathic disorders. J. Neurol. Neurosurg. Psychiatry.

[21] Head S.I., Williams D.A., Stephenson D.G. (1992). Abnormalities in structure and function of limb skeletal muscle fibres of dystrophic mdx mice. Proc. Biol. Sci..

[22] Blaivas M., Carlson B.M. (1991). Muscle fiber branching—Difference between grafts in old and young rats. Mech. Ageing Dev..

[23] Bourke D.L., Ontell M. (1986). Modification of the phenotypic expression of murine dystrophy: A morphological study. Anat. Rec..

[24] Tamaki T., Akatsuka A., Uchiyama S., Uchiyama Y., Shiraishi T. (1997). Appearance of complex branched muscle fibers is associated with a shift to slow muscle characteristics. Acta Anat..

[25] Zádor E., Mendler L., Takács V., de Bleecker J., Wuytack F. (2001). Regenerating soleus and extensor digitorum longus muscles of the rat show elevated levels of TNF-alpha and its receptors, TNFR-60 and TNFR-80. Muscle Nerve.

[26] Murach K.A., Dungan C.M., Peterson C.A., McCarthy J.J. (2019). Muscle Fiber Splitting Is a Physiological Response to Extreme Loading in Animals. Exerc. Sport Sci. Rev..

[27] Antonio J., Gonyea W.J. (1993). Progressive stretch overload of skeletal muscle results in hypertrophy before hyperplasia. J. Appl. Physiol..

[28] Antonio J., Gonyea W.J. (1994). Muscle fiber splitting in stretch-enlarged avian muscle. Med. Sci. Sports Exerc..

[29] Ho K.W., Roy R.R., Tweedle C.D., Heusner W.W., Van Huss W.D., Carrow R.E. (1980). Skeletal muscle fiber splitting with weight-lifting exercise in rats. Am. J. Anat..

[30] Tamaki T., Uchiyama S., Nakano S. (1992). A weight-lifting exercise model for inducing hypertrophy in the hindlimb muscles of rats. Med. Sci. Sports Exerc..

[31] Gonyea W., Ericson G.C., Bonde-Petersen F. (1977). Skeletal muscle fiber splitting induced by weight-lifting exercise in cats. Acta Physiol. Scand..

[32] Gonyea W.J., Sale D.G., Gonyea F.B., Mikesky A. (1986). Exercise induced increases in muscle fiber number. Eur. J. Appl. Physiol. Occup. Physiol..

[33] Faber R.M., Hall J.K., Chamberlain J.S., Banks G.B. (2014). Myofiber branching rather than myofiber hyperplasia contributes to muscle hypertrophy in mdx mice. Skelet. Muscle.

[34] Partridge T.A. (2021). Enhancing Interrogation of Skeletal Muscle Samples for Informative Quantitative Data. J. Neuromuscul. Dis..

[35] Goldberg A. (1967). Work-induced growth of skeletal muscle in normal and hypophysectomized rats. Am. J. Physiol.-Leg. Content.

[36] Hall-Craggs E.C. (1970). The longitudinal division of fibres in overloaded rat skeletal muscle. J. Anat..

[37] Kirby T.J., McCarthy J.J., Peterson C.A., Fry C.S. (2016). Synergist Ablation as a Rodent Model to Study Satellite Cell Dynamics in Adult Skeletal Muscle. Methods Mol. Biol..

[38] Højfeldt G., Sorenson T., Gonzales A., Kjaer M., Andersen J.L., Mackey A.L. (2023). Fusion of myofibre branches is a physiological feature of healthy human skeletal muscle regeneration. Skelet. Muscle.

[39] Harris J.B. (2003). Myotoxic phospholipases A2 and the regeneration of skeletal muscles. Toxicon.

[40] Head S.I., Houweling P.J., Chan S., Chen G., Hardeman E.C. (2014). Properties of regenerated mouse extensor digitorum longus muscle following notexin injury. Exp. Physiol..

[41] Dixon R.W., Harris J.B. (1996). Myotoxic activity of the toxic phospholipase, notexin, from the venom of the Australian tiger snake. J. Neuropathol. Exp. Neurol..

[42] Griffin C.A., Kafadar K.A., Pavlath G.K. (2009). MOR23 Promotes Muscle Regeneration and Regulates Cell Adhesion and Migration. Dev. Cell.

[43] Plant D.R., Colarossi F.E., Lynch G.S. (2006). Notexin causes greater myotoxic damage and slower functional repair in mouse skeletal muscles than bupivacaine. Muscle Nerve.

[44] Tamaki T., Akatsuka A. (1994). Appearance of complex branched fibers following repetitive muscle trauma in normal rat skeletal muscle. Anat. Rec..

[45] Buttgereit A., Weber C., Garbe C.S., Friedrich O. (2013). From chaos to split-ups-SHG microscopy reveals a specific remodelling mechanism in ageing dystrophic muscle. J. Pathol..

[46] Cooper S.T., Head S.I. (2014). Membrane Injury and Repair in the Muscular Dystrophies. Neuroscientist.

[47] Chan S., Head S.I., Morley J.W. (2007). Branched fibers in dystrophic mdx muscle are associated with a loss of force following lengthening contractions. Am. J. Physiol. Cell Physiol..

[48] Duddy W., Duguez S., Johnston H., Cohen T.V., Phadke A., Gordish-Dressman H., Nagaraju K., Gnocchi V., Low S., Partridge T. (2015). Muscular dystrophy in the mdx mouse is a severe myopathy compounded by hypotrophy, hypertrophy and hyperplasia. Skelet. Muscle.

[49] Lovering R.M., Michaelson L., Ward C.W. (2009). Malformed mdx myofibers have normal cytoskeletal architecture yet altered EC coupling and stress-induced Ca^2+^ signaling. Am. J. Physiol. Cell Physiol..

[50] Tamaki T., Sekine T., Akatsuka A., Uchiyama S., Nakano S. (1993). Three-dimensional cytoarchitecture of complex branched fibers in soleus muscle from mdx mutant mice. Anat. Rec..

[51] Henry C.C., Martin K.S., Ward B.B., Handsfield G.G., Peirce S.M., Blemker S.S. (2017). Spatial and age-related changes in the microstructure of dystrophic and healthy diaphragms. PLoS ONE.

[52] Järvinen T.A., Järvinen T.L., Kääriäinen M., Kalimo H., Järvinen M. (2005). Muscle injuries: Biology and treatment. Am. J. Sports Med..

[53] Papadimitriou J.M., Robertson T.A., Mitchell C.A., Grounds M.D. (1990). The process of new plasmalemma formation in focally injured skeletal muscle fibers. J. Struct. Biol..

[54] Kiriaev L., Kueh S., Morley J.W., North K.N., Houweling P.J., Head S.I. (2021). Lifespan Analysis of Dystrophic mdx Fast-Twitch Muscle Morphology and Its Impact on Contractile Function. Front. Physiol..

[55] Carlson C.G. (2022). Does the Pathogenic Sequence of Skeletal Muscle Degeneration in Duchenne Muscular Dystrophy Begin and End with Unrestrained Satellite Cell Activation?. Muscles.

[56] Allen D.G., Whitehead N.P., Froehner S.C. (2016). Absence of Dystrophin Disrupts Skeletal Muscle Signaling: Roles of Ca^2+^, Reactive Oxygen Species, and Nitric Oxide in the Development of Muscular Dystrophy. Physiol. Rev..

[57] Siegel A.S., Henley S., Zimmerman A., Miles M., Plummer R., Kurz J., Balch F., Rhodes J.A., Shinn G.L., Carlson C.G. (2011). The influence of passive stretch and NF-κB inhibitors on the morphology of dystrophic muscle fibers. Anat. Rec..

[58] Nielsen C., Potter R.M., Borowy C., Jacinto K., Kumar R., Carlson C.G. (2017). Postnatal Hyperplasic Effects of ActRIIB Blockade in a Severely Dystrophic Muscle. J. Cell. Physiol..

[59] Bockhold K.J., David Rosenblatt J., Partridge T.A. (1998). Aging normal and dystrophic mouse muscle: Analysis of myogenicity in cultures of living single fibers. Muscle Nerve.

[60] Kiriaev L., Kueh S., Morley J.W., North K.N., Houweling P.J., Head S.I. (2018). Branched fibers from old fast-twitch dystrophic muscles are the sites of terminal damage in muscular dystrophy. Am. J. Physiol. Cell Physiol..

[61] Pavlath G.K. (2010). A new function for odorant receptors. Cell Adh. Migr..

[62] Patel T.J., Lieber R.L. (1997). Force transmission in skeletal muscle: From actomyosin to external tendons. Exerc. Sport Sci. Rev..

[63] Loeb G.E., Pratt C.A., Chanaud C.M., Richmond F.J.R. (1987). Distribution and innervation of short, interdigitated muscle fibers in parallel-fibered muscles of the cat hindlimb. J. Morphol..

[64] Ounjian M., Roy R.R., Eldred E., Garfinkel A., Payne J.R., Armstrong A., Toga A.W., Edgerton V.R. (1991). Physiological and developmental implications of motor unit anatomy. J. Neurobiol..

[65] Lynch G.S., Hinkle R.T., Chamberlain J.S., Brooks S.V., Faulkner J.A. (2001). Force and power output of fast and slow skeletal muscles from mdx mice 6-28 months old. J. Physiol..

[66] Massopust R.T., Lee Y.I., Pritchard A.L., Nguyen V.M., McCreedy D.A., Thompson W.J. (2020). Lifetime analysis of mdx skeletal muscle reveals a progressive pathology that leads to myofiber loss. Sci. Rep..

[67] Young M., Paul A., Rodda J., Duxson M., Sheard P. (2000). Examination of intrafascicular muscle fiber terminations: Implications for tension delivery in series-fibered muscles. J. Morphol..

[68] Karpati G., Carpenter S., Prescott S. (1988). Small-caliber skeletal muscle fibers do not suffer necrosis in mdx mouse dystrophy. Muscle Nerve.

[69] Petrof B.J., Shrager J.B., Stedman H.H., Kelly A.M., Sweeney H.L. (1993). Dystrophin protects the sarcolemma from stresses developed during muscle contraction. Proc. Natl. Acad. Sci. USA.

[70] Petrof B.J. (2002). Molecular pathophysiology of myofiber injury in deficiencies of the dystrophin-glycoprotein complex. Am. J. Phys. Med. Rehabil..

[71] Lindsay A., Baumann C.W., Rebbeck R.T., Yuen S.L., Southern W.M., Hodges J.S., Cornea R.L., Thomas D.D., Ervasti J.M., Lowe D.A. (2020). Mechanical factors tune the sensitivity of mdx muscle to eccentric strength loss and its protection by antioxidant and calcium modulators. Skelet. Muscle.

[72] Brooks S.V., Zerba E., Faulkner J.A. (1995). Injury to muscle fibres after single stretches of passive and maximally stimulated muscles in mice. J. Physiol..

[73] Coulton G.R., Curtin N.A., Morgan J.E., Partridge T.A. (1988). The mdx mouse skeletal muscle myopathy: II. contractile properties. Neuropathol. Appl. Neurobiol..

[74] Dellorusso C., Crawford R.W., Chamberlain J.S., Brooks S.V. (2001). Tibialis anterior muscles in mdx mice are highly susceptible to contraction-induced injury. J. Muscle Res. Cell Motil..

[75] Froehner S.C., Reed S.M., Anderson K.N., Huang P.L., Percival J.M. (2014). Loss of nNOS inhibits compensatory muscle hypertrophy and exacerbates inflammation and eccentric contraction-induced damage in mdx mice. Hum. Mol. Genet..

[76] Hakim C.H., Duan D. (2012). Gender differences in contractile and passive properties of mdx extensor digitorum longus muscle. Muscle Nerve.

[77] Hakim C.H., Grange R.W., Duan D. (2011). The passive mechanical properties of the extensor digitorum longus muscle are compromised in 2- to 20-mo-old mdx mice. J. Appl. Physiol. (1985).

[78] Pastoret C., Sebille A. (1993). Time course study of the isometric contractile properties of mdx mouse striated muscles. J. Muscle Res. Cell Motil..

[79] Pastoret C., Sebille A. (1995). Mdx mice show progressive weakness and muscle deterioration with age. J. Neurol. Sci..

[80] Quinlan J.G., Johnson S.R., McKee M.K., Lyden S.P. (1992). Twitch and tetanus in mdx mouse muscle. Muscle Nerve.

[81] Wernig A., Irintchev A., Weisshaupt P. (1990). Muscle injury, cross-sectional area and fibre type distribution in mouse soleus after intermittent wheel-running. J. Physiol..

[82] Brooks S.V. (1998). Rapid recovery following contraction-induced injury to in situ skeletal muscles in mdx mice. J. Muscle Res. Cell Motil..

[83] Faulkner J.A., Brooks S.V., Dennis R.G., Lynch G.S. (1997). The functional status of dystrophic muscles and functional recovery by skeletal muscles following myoblast transfer. BAM-PADOVA-.

[84] Han R., Rader E.P., Levy J.R., Bansal D., Campbell K.P. (2011). Dystrophin deficiency exacerbates skeletal muscle pathology in dysferlin-null mice. Skelet. Muscle.

[85] Lindsay A., Schmiechen A., Chamberlain C.M., Ervasti J.M., Lowe D.A. (2018). Neopterin/7,8-dihydroneopterin is elevated in Duchenne muscular dystrophy patients and protects mdx skeletal muscle function. Exp. Physiol..

[86] Williams D.A., Head S.I., Lynch G.S., Stephenson D.G. (1993). Contractile properties of skinned muscle fibres from young and adult normal and dystrophic (mdx) mice. J. Physiol..

[87] Friedrich O., Both M., Weber C., Schurmann S., Teichmann M.D., von Wegner F., Fink R.H., Vogel M., Chamberlain J.S., Garbe C. (2010). Microarchitecture is severely compromised but motor protein function is preserved in dystrophic mdx skeletal muscle. Biophys. J..

[88] Head S.I. (2010). Branched fibres in old dystrophic mdx muscle are associated with mechanical weakening of the sarcolemma, abnormal Ca2+ transients and a breakdown of Ca^2+^ homeostasis during fatigue. Exp. Physiol..

[89] Brown L.M., Lopez J.R., Olsen J.A., Rüdel R., Simmons R.M., Taylor S.R., Wanek L.A. (1982). Branched skeletal muscle fibers not associated with dysfunction. Muscle Nerve.

[90] Goldstein S.S., Rall W. (1974). Changes of action potential shape and velocity for changing core conductor geometry. Biophys. J..

[91] Joyner R.W., Westerfield M., Moore J.W. (1980). Effects of cellular geometry on current flow during a propagated action potential. Biophys. J..

[92] Iyer S.R., Shah S.B., Valencia A.P., Schneider M.F., Hernandez-Ochoa E.O., Stains J.P., Blemker S.S., Lovering R.M. (2017). Altered nuclear dynamics in MDX myofibers. J. Appl. Physiol. (1985).

[93] Goodall M.H., Ward C.W., Pratt S.J.P., Bloch R.J., Lovering R.M. (2012). Structural and functional evaluation of branched myofibers lacking intermediate filaments. Am. J. physiology. Cell Physiol..

[94] Hernández-Ochoa E.O., Pratt S.J.P., Garcia-Pelagio K.P., Schneider M.F., Lovering R.M. (2015). Disruption of action potential and calcium signaling properties in malformed myofibers from dystrophin-deficient mice. Physiol. Rep..

[95] Lieber R. (2011). Skeletal Muscle Structure, Function, and Plasticity.

[96] Lieber R.L., Woodburn T.M., Friden J. (1991). Muscle damage induced by eccentric contractions of 25% strain. J. Appl. Physiol..

[97] Morgan J., Partridge T. (2020). Skeletal muscle in health and disease. Dis. Model. Mech..

[98] Zhang B.-T., Whitehead N.P., Gervasio O.L., Reardon T.F., Vale M., Fatkin D., Dietrich A., Yeung E.W., Allen D.G. (2012). Pathways of Ca^2+^ entry and cytoskeletal damage following eccentric contractions in mouse skeletal muscle. J. Appl. Physiol..

[99] Janghra N., Morgan J.E., Sewry C.A., Wilson F.X., Davies K.E., Muntoni F., Tinsley J. (2016). Correlation of Utrophin Levels with the Dystrophin Protein Complex and Muscle Fibre Regeneration in Duchenne and Becker Muscular Dystrophy Muscle Biopsies. PLoS ONE.

[100] Evans W.J., Hellerstein M., Butterfield R.J., Smith E., Guglieri M., Katz N., Nave B., Branigan L., Thera S., Vordos K.L. (2024). Reductions in functional muscle mass and ability to ambulate in Duchenne muscular dystrophy from ages 4 to 24 years. J. Physiol..

[101] Stedman H.H., Sweeney H.L., Shrager J.B., Maguire H.C., Panettieri R.A., Petrof B., Narusawa M., Leferovich J.M., Sladky J.T., Kelly A.M. (1991). The mdx mouse diaphragm reproduces the degenerative changes of Duchenne muscular dystrophy. Nature.

[102] Yeung E.W., Head S.I., Allen D.G. (2003). Gadolinium reduces short-term stretch-induced muscle damage in isolated mdx mouse muscle fibres. J. Physiol..

[103] Yeung E.W., Whitehead N.P., Suchyna T.M., Gottlieb P.A., Sachs F., Allen D.G. (2005). Effects of stretch-activated channel blockers on [Ca^2+^]i and muscle damage in the mdx mouse. J. Physiol..

[104] Khairallah R.J., Shi G., Sbrana F., Prosser B.L., Borroto C., Mazaitis M.J., Hoffman E.P., Mahurkar A., Sachs F., Sun Y. (2012). Microtubules Underlie Dysfunction in Duchenne Muscular Dystrophy. Sci. Signal..

[105] Olthoff J.T., Lindsay A., Abo-Zahrah R., Baltgalvis K.A., Patrinostro X., Belanto J.J., Yu D.Y., Perrin B.J., Garry D.J., Rodney G.G. (2018). Loss of peroxiredoxin-2 exacerbates eccentric contraction-induced force loss in dystrophin-deficient muscle. Nat. Commun..

[106] Call J.A., Warren G.L., Verma M., Lowe D.A. (2013). Acute failure of action potential conduction in *mdx* muscle reveals new mechanism of contraction-induced force loss. J. Physiol..

[107] Roy P., Rau F., Ochala J., Messéant J., Fraysse B., Lainé J., Agbulut O., Butler-Browne G., Furling D., Ferry A. (2016). Dystrophin restoration therapy improves both the reduced excitability and the force drop induced by lengthening contractions in dystrophic mdx skeletal muscle. Skelet. Muscle.

[108] Capogrosso R.F., Mantuano P., Cozzoli A., Sanarica F., Massari A.M., Conte E., Fonzino A., Giustino A., Rolland J.F., Quaranta A. (2017). Contractile efficiency of dystrophic mdx mouse muscle: In vivo and ex vivo assessment of adaptation to exercise of functional end points. J. Appl. Physiol. (1985).

[109] Moens P., Baatsen P.H.W.W., Marechal G. (1993). Increased susceptibility of EDL muscles from mdx mice to damage induced by contractions with stretch. J. Muscle Res. Cell Motil..

[110] Pichavant C., Burkholder T.J., Pavlath G.K. (2015). Decrease of myofiber branching via muscle-specific expression of the olfactory receptor mOR23 in dystrophic muscle leads to protection against mechanical stress. Skelet. Muscle.

[111] Zanou N., Gailly P. (2013). Skeletal muscle hypertrophy and regeneration: Interplay between the myogenic regulatory factors (MRFs) and insulin-like growth factors (IGFs) pathways. Cell. Mol. Life Sci..

[112] Di Filippo E.S., Mancinelli R., Marrone M., Doria C., Verratti V., Toniolo L., Dantas J.L., Fulle S., Pietrangelo T. (2017). Neuromuscular electrical stimulation improves skeletal muscle regeneration through satellite cell fusion with myofibers in healthy elderly subjects. J. Appl. Physiol. (1985).

[113] Selsby J.T., Morine K.J., Pendrak K., Barton E.R., Sweeney H.L. (2012). Rescue of dystrophic skeletal muscle by PGC-1alpha involves a fast to slow fiber type shift in the mdx mouse. PLoS ONE.

[114] Russell A.J., DuVall M., Barthel B., Qian Y., Peter A.K., Newell-Stamper B.L., Hunt K., Lehman S., Madden M., Schlachter S. (2023). Modulating fast skeletal muscle contraction protects skeletal muscle in animal models of Duchenne muscular dystrophy. J. Clin. Investig..

[115] Lindsay A., Southern W.M., McCourt P.M., Larson A.A., Hodges J.S., Lowe D.A., Ervasti J.M. (2019). Variable cytoplasmic actin expression impacts the sensitivity of different dystrophin-deficient mdx skeletal muscles to eccentric contraction. FEBS J..

[116] Kiriaev L., Kueh S., Morley J.W., Houweling P.J., Chan S., North K.N., Head S.I. (2021). Dystrophin-negative slow-twitch soleus muscles are not susceptible to eccentric contraction induced injury over the lifespan of the mdx mouse. Am. J. Physiol. Cell Physiol..

